# Supplementary material for: Mapping the Implementation of a Clinical Pharmacist-Driven Antimicrobial Stewardship Programme at a Tertiary Care Centre in South India
Source: Antibiotics (Basel). 2021 Feb 23;10(2):220. doi: 10.3390/antibiotics10020220 (PMC7926893; doi:10.3390/antibiotics10020220)
Supplement: Supplementary file 1 [file antibiotics-10-00220-s001.zip › Supplementary data- AMSP recommendation form.docx]

| 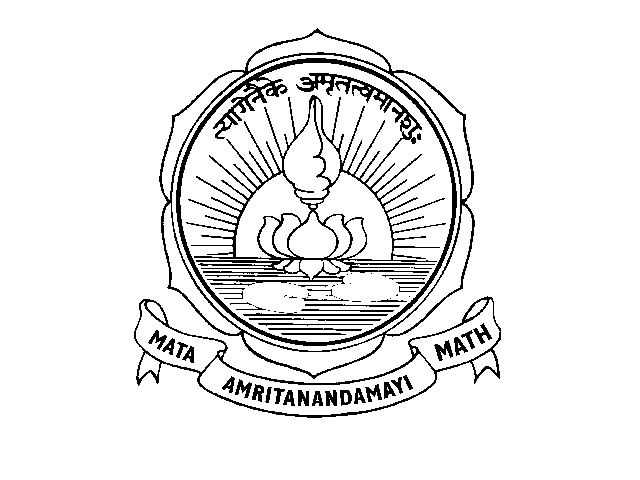 | **Amrita Institute of Medical Sciences and Research Centre** (an ISO 9001/14001/18001/NABH/NABL/NAAC certified hospital)  **ANTIBIOTIC STEWARDSHIP COMMITTEE** |
| --- | --- |

**RECOMMENDATION FORM**

**Dear Doctor:**

**The following information is presented to you for your review and evaluation regarding this patient’s antimicrobial therapy. These recommendations are based primarily on laboratory data and your clinical judgment of the patient’s condition should be used to determine the best treatment course for the patient.**

**Allergies: _____________ Patient’s Name: _________________________ MRD No: _______________Room:________**

**Current Culture and Sensitivity Data**

| **Date** | **Source** | **Culture and Sensitivity result** |
| --- | --- | --- |
|  |  |  |
|  |  |  |
|  |  |  |
|  |  |  |
|  |  |  |
|  |  |  |
|  |  |  |

**Current Antibiotic Regimen:**

| **Date** | **Antibiotic** | **Days of Therapy** |
| --- | --- | --- |
|  |  |  |
|  |  |  |
|  |  |  |
|  |  |  |
|  |  |  |
|  |  |  |

**Clinical Data:**

| **Date** | **Data** | **Findings** |
| --- | --- | --- |
|  |  |  |
|  |  |  |
|  |  |  |
|  |  |  |

**Please consider the following change(s) to the antibiotic regimen:**

**1.**

**2.**

**3.**

**4.**

**5.**

If infection has been ruled out, please consider discontinuing antimicrobial therapy

**Antibiotic Stewardship team**

**Dr.Sanjeev K Singh, Dr.Vidya P Menon, Dr.Zubair, Dr.Anil Kumar Dr.Shyam, ,Dr.Vrinda, Dr.Sangita,Dr.Ananya**

NOTE: THIS IS NOT A PART OF THE PATIENT’S PERMANENT MEDICAL RECORD
