# Supplementary material for: Mapping the Implementation of a Clinical Pharmacist-Driven Antimicrobial Stewardship Programme at a Tertiary Care Centre in South India
Source: Antibiotics (Basel). 2021 Feb 23;10(2):220. doi: 10.3390/antibiotics10020220 (PMC7926893; doi:10.3390/antibiotics10020220)
Supplement: Supplementary file 1 [file antibiotics-10-00220-s001.zip › Supplementary data- AMSP CRF.docx]

| \| 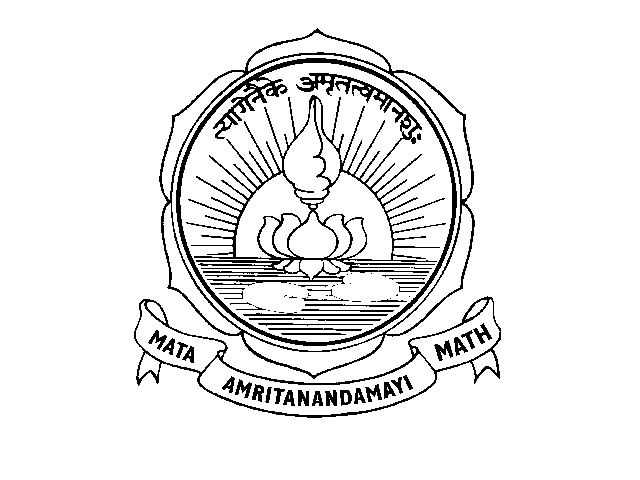 \| **Amrita Institute of Medical Sciences and Research Centre** (an ISO 9001/14001/18001/NABH/NABL/NAAC certified hospital)  **ANTIBIOTIC STEWARDSHIP COMMITTEE** \| \| --- \| --- \|   **Data Collection Form**  1.Name of the patient  2.MRD No:  3.Date of Admission 4.Date of Review  5.Age in years 6.Sex:Male/Female  7.Location  8.Admitting Doctor  9.Admission Diagnosis  10. Suspected focus of infection   1. Pneumonia 2. UTI 3. CNS 4. Skin & Soft Tissue 5. Abdominal 6. Bacteremia 7. Catheter/Lines/Stents 8. Other:   11.Cultures   1. Culture sent- Yes / No 2. Date and time of culture sent : 3. Sample sent for culture   a. Blood  b. Urine  c. Stool  d. Sputum  e. Mini Bal  f.CSF  g. Ascetic fluid  h. Pleural fluid  i. Tissue  j. Pus    D) Provisional report of culture - after 48 hours of sending ( To include culture and sensitivity report if available)  12) S .Creatinine(mg/dl)    13) Antibiotics used   \| Antibiotic \| Dose \| Route \| Frequency \| Date of initiation \| Loading dose \| Infusion \| \| --- \| --- \| --- \| --- \| --- \| --- \| --- \| \|  \|  \|  \|  \|  \|  \|  \| \|  \|  \|  \|  \|  \|  \|  \| \|  \|  \|  \|  \|  \|  \|  \| \|  \|  \|  \|  \|  \|  \|  \| \|  \|  \|  \|  \|  \|  \|  \|       14) Clinical Signs correlating with Antibiotic initiation(prior 48 hours)  Temp(^0^F)-  BP(mmHg)-  RR(per minute)-  O2 saturation (%)-  WBC(K/uL)-  CRP(mg/L)-  Procalcitonin (ng/ml)-  Lactate(mmol/L) -  At 48 hours  Antibiotic changed |
| --- | --- | --- | --- | --- | --- | --- | --- | --- | --- | --- | --- | --- | --- | --- | --- | --- | --- | --- | --- | --- | --- | --- | --- | --- | --- | --- | --- | --- | --- | --- | --- | --- | --- | --- | --- | --- | --- | --- | --- | --- | --- | --- | --- | --- |
|  |
